# Effects of Enriched Charcoal as Permanent 0.2% Feed-Additive in Standard and Low-Protein Diets of Male Fattening Turkeys: An On-Farm Study

**DOI:** 10.3390/ani9080541

**Published:** 2019-08-08

**Authors:** Katharina Hinz, Jenny Stracke, Jule Katrin Schättler, Nicole Kemper, Birgit Spindler

**Affiliations:** 1 Institute for Animal Hygiene, Animal Welfare and Farm Animal Behaviour, University of Veterinary Medicine Hannover, Foundation, Bischofsholer Damm 15 (Building 116), D-30173 Hannover, Germany; 2 Chamber of Agriculture Lower Saxony, Division Agriculture, Mars-la-Tour-Str. 6, D-26121 Oldenburg, Germany

**Keywords:** turkey, footpad dermatitis, charcoal, crude protein reduced diet, compensatory growth

## Abstract

**Simple Summary:**

Improving animal welfare, animal health and environmental issues is important in modern animal farming. In fattening of male turkeys, footpad dermatitis is a very frequent problem. Reducing faecal moisture is one way of controlling one source of footpad dermatitis. Therefore, in the present study, conducted under on-farm conditions, we added 0.2% enriched charcoal (known as a household remedy in therapy of diarrhoea and microbial imbalances) to the diet. Additionally, in a second experiment, the protein content of the diet was reduced by 1% from weeks 6–13 of life to slow down the growth rate and to reduce nitrogen in the litter. Three farms were visited four times during the fattening period to collect data on the birds. The charcoal-supplemented diet showed no effects on the performance of the birds or the examined health parameters. The protein-reduced and charcoal-supplemented diet did not influence the final bodyweight or the footpad status but reduced the mortality during the fattening period by 0.5%. In conclusion, enriched charcoal as 0.2% feed additive does not improve animal health, welfare or performance. Hence, a diet with temporarily reduced protein shows beneficial effects on the mortality rate and has no negative influence on the final body weight.

**Abstract:**

Wet litter is the most important cause of footpad dermatitis in poultry, this in turn being a highly relevant animal-related welfare indicator. This field study was subdivided into two experiments. In Experiment 1, the standard diet was supplemented by 0.2% enriched charcoal, being a non-specific absorber and therefore might be promising in reducing faecal moisture. In Experiment 2, the experimental group received a reduced crude protein diet during weeks 6–13, combined with a 0.2% enriched charcoal supplementation. The trials were each conducted with two batches on three farms under on-farm conditions. The animals were observed at 6, 10, 14 and 18 weeks of age to collect data on body weight and different health parameters. The mortality and litter samples were analysed after slaughtering. In Experiment 1, performance and health were not affected despite higher dry matter content of the litter. In Experiment 2, the weight of birds receiving the protein-reduced diet was decreased significantly throughout the experiment. However, the slaughter weight did not differ. The mortality was reduced by 0.5% in the experimental group. Therefore, it was concluded that 0.2% of enriched charcoal is not a valuable feed-additive regarding animal health, while temporary protein reduction might have positive effects.

## 1. Introduction

In modern animal farming, it is important to improve management to facilitate animal welfare. Wet litter is the highest risk factor for the development of footpad dermatitis in turkeys [1,2]. Feeding enriched charcoal could be an option to bind liquids in the faeces, which consequently might lead to a drier litter surface. Charcoal is a non-specific absorber that can be produced by the pyrolysis of wood or other high carbon materials [3]. In the process, a porous structure is formed, creating a large inner surface, which is the basis for its absorbing characteristics [4,5]. Therefore, it is generally considered helpful in diarrhoea or intoxication cases in humans [6] as well as in animals [7]. There are many studies demonstrating its benefits in enhancing growth performance, feed efficiency or morphological intestinal parameters such as villus height in fish [8,9], pigs [10,11,12,13] and poultry [14,15,16,17,18,19]. Furthermore, carbon is able to adsorb microorganisms, for example *Escherichia coli* [20] and *Salmonella* species [19,21,22] and helps in the treatment of *Cryptosporidium parvum* in goat kids and calves [23,24]. As *Escherichia coli* or *Clostridium perfringens* are some of the most important reasons for antibiotic medication in turkeys, charcoal might therefore improve livestock intestinal health overall [25,26]. However, results are inconsistent, as other studies find no such effects of charcoal [17]. In turkeys, there is little knowledge about the effects of dietary charcoal. Majewska et al. [27] found 0.3% charcoal to have a positive impact on growth performance, feed conversion ratio, mortality and crude protein content of the breast muscle; these findings were confirmed by Majewska et al. [28].

A reduction in the crude protein content in the feed might also have a positive effect on the footpad health of turkeys. In broiler chickens, such reduced crude protein diets are known to slow down the animals’ growth rate and can reduce the severity of footpad dermatitis [29]. Furthermore, reducing the amount of crude protein in the diet might help to reduce nitrogen emission. This topic is currently acquiring a higher relevance, especially in areas with high-density farming like in Northern Germany. Over-fertilisation with manure from litter can lead to nitrates in groundwater, bearing health risks, such as human intestine colorectal cancer [30]. However, in turkeys, protein-reduced diets are only rarely used in practice due to concerns of economic losses caused by lower slaughter weights [31,32,33]. In the present study, crude protein was reduced temporarily to take advantage of the compensatory growth after a period of undernutrition as described by Auckland and Morris [34] in turkeys and by Zubair and Leeson [35] in broiler chickens. The aim of the present study was to investigate the effect of herbal fermentation extract (FKE) enriched charcoal as 0.2% permanent supplement to the standard diet and to a temporary 1% crude protein reduced diet from the 6th to the 14th week of life in male turkeys during the fattening period.

## 2. Material and Methods

All of the animals were housed in accordance with EU (European Directive 2008/120/EC), national law (Tierschutzgesetz, Tierschutz-Nutztierhaltungs-Verordnung) and national guidlines (Bundeseinheitliche Eckwerte für eine freiwillige Vereinbarung zur Haltung von Mastputen). In compliance with European Directive 2010/63/EC Article 1 5. (f), the present study did not imply any invasive procedure or treatment to the animals. The authors declare that the study was in accordance with current German law. This study was reviewed and it received approval from the Animal Welfare Officer of the University of Veterinary Medicine Hannover, Foundation (TVO-2017-V-122).

### 2.1. Birds and Housing

For the experiments, large male white turkeys (95000 B.U.T. 6, 9900 B.U.T. TP7; Aviagen^®^, Huntsville, AL, USA) were kept on three commercial farms in Northern Germany from January 2017 to October 2018. Each farm provided two identical stables equipped with a ventilation system controlled by flaps or curtains. Therefore, each farm kept the control and treatment flocks simultaneously. The stables housed 3500 to 4950 animals during the fattening period. Two farms needed to comply with the regulations of Initiative Tierwohl, a German programme promoting farm animal welfare, so the stocking density was limited to 53 kg/m^2^. Furthermore, per 400 m^2^ at least two materials for manipulating were offered such as square bales of straw and stones for pecking [36]. The stocking density on the third farm was limited to 58 kg/m^2^, complying with the regulations of Bundeseinheitliche Eckwerte für eine freiwillige Vereinbarung zur Haltung von Mastputen (Federal Uniform Benchmark Figures for a Voluntary Agreement on the Keeping of Fattening Turkeys) [37]. Per 400 m^2^, at least one material for manipulating was offered (detailed information is shown in Table A1). New materials were provided in the case of increased feather pecking and cannibalism. On every farm, long-stalked straw was used as bedding and new litter was given every second to third day in both stables in equal amounts. Spray cooling systems and additional ventilators were used for cooling the air on days with high temperatures. The pens were brightened by natural daylight. If necessary, artificial light was switched on abiding by a dark period of 8 hours. The turkeys were all vaccinated against Newcastle disease, haemorrhagic enteritis virus (HEV), an acute viral disease in young turkeys, turkey rhinotracheitis and *Ornithobacterium rhinotracheale*. Additionally, the farms administered vaccines against Avian Influenza Virus, *Mycoplasma synoviae*, avian reovirus infection, *Escherichia coli*, *Staphylococcus aureus* and flock-specific vaccines depending on their individual management. The date of entry to the fattening stables varied between the fourth to the fifth week of life; the experiments started with the third feeding phase on day 36 of life. For the second experiment on Farm 2, the treatment started already in the rearing stables because the turkey toms were moved later to the fattening barns in the sixth and eighth week of life. All birds were slaughtered at 147 ± 4 days of life.

### 2.2. Experimental Design

The turkeys in the control groups on the three farms were fed with a standard six-phase system diet (BEST 3 Geflügelernährung GmbH, Twistringen, Germany). The nutrient content of the diets is shown in Table 1 and the formulae are provided in Table 2.

#### 2.2.1. Experiment 1

For the first experiment, the feed of the experimental flocks of the three farms (*n* = 6 fattening periods) was supplemented by 0.2% powdered charcoal (Phyto Carbon GmbH, Hünxe, Germany; characteristics shown in Table 3) using a special dosing unit from phase three up to slaughtering, while the control flocks (*n* = 6 fattening periods) received the pure standard diet. The coal was carbonised from mixed non-manufactured wood originating from regional forests and sprayed with FKE (Fermentierter Kräuterextrakt, an extract from fermented herbs containing organic acids and microorganisms, from multikraft^®^, Multikraft Produktions-und HandelsgmbH, Pichl/Wels, Austria).

#### 2.2.2. Experiment 2

In the second experiment, the treatment flock of the three farms (*n* = 6 fattening periods) received a diet with 1% less crude protein during phases three and four by admixing wheat; in phases five and six they received the standard diet. Furthermore, the diet in this group was supplemented by 0.2% powdered charcoal from phase three up to slaughtering. The control group of each farm was fed the pure standard diet. On Farm 3, a feed from another company (GS agri eG, Schneiderkrug, Germany) was given to the second batch of animals in the second experiment, but the nutrient content thereof was equalised.

### 2.3. Data Acquisition

The farms were visited every 4 weeks when the turkeys were 6, 10, 14 and 18 weeks of age. At every point in time, the turkeys (n = 40) were weighed with poultry scales (manual poultry scales BAT1, VEIT Electronics, Moravany, the Czech Republic). Furthermore, the status of the footpads of the metatarsus, breast skin and faecal soiling of the cloacae were assessed. In the case of the footpads, each foot was scored separately. For data analysis, only one score per bird was assigned, grading the worst lesion of both feet. In animals with more than one breast button, only the most severe alteration was recorded. The scoring systems and their description can be found in Table 4. The scoring data of the first observation date of Experiment 1 in Batch 1 on Farm 2 are missing due to technical problems. Furthermore, the data of two turkeys from the first examination date of Experiment 1 in Batch 1 are missing as well due to technical problems. Data on medication, mortality and feed consumption were registered when possible; data of Farm 3 are missing due to technical problems. Furthermore, the consistency of fresh faeces (n = 40) was scored visually (scores shown in Table 4). After turkeys were removed from the barns for slaughtering, a pooled sample of litter was collected, and dry matter content and total nitrogen content were analysed by the LUFA Nord-West, Institute for Fertilisers and Seeds, Hamelin, Germany in accordance with the methods of DIN ISO 11261; 1997-05 and of DIN EN 12880-S 2a; 2001-02, respectively.

### 2.4. Statistical Analyses

Statistical analysis was conducted using SAS Version 9.4 (SAS Institute Inc., Cary, NC, USA). After testing for normality using a normal plot, the body weight data were analysed with a mixed linear model (MIXED procedure). Diet (Experiment 1: charcoal versus standard; Experiment 2: charcoal + reduced protein versus standard), farm (1–3) and the interactions between treatment and farm were included as fixed factors. For Experiment 2, the fixed factors also contained the age and the interaction between treatment and age. The random effects comprised the individual animal (1–40), nested according to age (6, 10, 14, 18 weeks) and nested according to batch (1, 2). Multiple pairwise comparisons were performed using Tukey–Kramer tests.

The data of dermatitis status of the footpads, breast skin and soiled cloaca were each analysed separately using the GENMOD procedure. Here again, diet (Experiment 1: charcoal versus standard; Experiment 2: charcoal + reduced protein versus standard), farm (1–3) and the interactions between treatment and farm were included as fixed factors. Random effects consisted of the individual animal (1–40), nested according to age (6, 10, 14, 18 weeks) and nested according to batch (1, 2). The DIST option and LINK function were set at DIST = multinomial and LINK = cumlogit. Mortality, data on feed intake, faecal quality and litter analysis were analysed using descriptive statistics.

The level of significance was set at *p* < 0.05.

## 3. Results

### 3.1. Experiment 1

#### 3.1.1. Performance

Data of the body weight were normally distributed. No effect of the charcoal was found (F = 0.26; *p* > 0.05). The farm revealed a significant effect (F = 241.52; *p* < 0.001), with pairwise comparisons showing a significant difference between each of the farms (all *t* > 6.6; all *p* < 0.001), though Farm 3 obtained the lowest weights. However, the interaction between farm and diet revealed no effect (F = 2.01; *p* > 0.05). The average mortality, average feed consumption and the number of medications administered during the fattening period did not differ between treatment groups. The dry matter content of the litter was on average 6.8 percentage points higher in the charcoal flocks than the control. The results are shown in Table 5.

#### 3.1.2. Health Status

Footpad dermatitis was observed from week 6 to 18 of life in high incidences (95.8% to 92.5%). Score 2 occurred most frequently during the entire fattening period varying in a range of 45.8% to 74.2% (shown in Table 6). The ratio of severe lesions, including Scores 3 and 4, increased from 11.2% to 34.1%. However, the diet had no significant effect on the footpad health (*p* > 0.05). The farm had a significant effect (*p* < 0.001) as did the interaction between farm and treatment (*p* < 0.001). Nonetheless, the effects of the farm followed no apparent patterns.

Breast buttons appeared mainly in the weeks 14 and 18 of age, only in a single batch did the first alterations occur earlier. The incidence was 26.9% on average at the last observation date; breast buttons with a diameter larger than 3 cm occurred at an incidence of 2.5%. A significant effect of the farm was detected (*p* < 0.001). Here again, the effects could not be traced back to any cause.

Soiled cloacae were detected from the first to the last date. However, in 6 week old turkeys, cloacae were soiled more frequently (12.5%) than in the older ones in week 18 of age (5%). The diet revealed no effect (*p* > 0.05). However, a significant effect of the farm was detected (*p* < 0.001). The quality of the faeces did not differ among the groups.

### 3.2. Experiment 2

#### 3.2.1. Performance

In the second experiment, the body weight was normally distributed as well. Here, the diet was found to have a significant effect (F = 5.29; *p* < 0.05), with animals treated with lower protein and charcoal revealing significantly lower weights than the control group. Furthermore, the farm revealed a significant effect (F = 510.47; *p* < 0.001), a comparison of all farms pairwise showing significant differences for all combinations (all *t* > 15.95; all *p* < 0.001). The age showed a significant effect (F = 46.77; *p* < 0.001), with higher weights increasing with age. However, no significant effect of the interaction between age and the treatment was found (F = 460.77; *p* > 0.05). In the flocks treated with the experimental diet, the mean mortality was lower by 0.55 percentage points compared to the control. The dry matter content of the litter was on average 2.2 percentage points higher in the flocks fed the experimental diet (shown in Table 7).

#### 3.2.2. Health Status

Footpad dermatitis occurred at an incidence of 82.8% on average at the beginning of the experiment. Feet with Score 0 or 1 dominated (71.2%). In the 18th week of life, the incidence was 98.1% on average with Score 2 being most frequent (56.5%). Severe lesions, complying with Scores 3 and 4, increased from 3.5% to 31.5% (shown in Table 8). The diet, however, had no significant effect on the food pad health (*p* > 0.05), whereas the farm and the interaction between the farm and treatment revealed an effect (*p* < 0.05). Nonetheless, clear patterns in these effects were not detected. In this experiment, breast blisters occurred sporadically in the 14th week of life and increased with age but did not exceed 12% on average. The diet revealed no significant effect (*p* > 0.05).

The occurrence of soiled cloacae was higher in the first half of the fattening period compared to the second half (up to 39% and 5%, respectively). The farm showed a significant effect (*p* < 0.05). Furthermore, a significant effect of the interaction between farm and diet was found (*p* < 0.05).

The quality of the faeces was better in the control flocks than in the treatment flocks at week 6 of life (72.5% and 55.8% Score 1, respectively). This difference reduced in the following weeks and did not appear in the second half of the fattening period.

## 4. Discussion

Feed supplements are given by farmers with the purpose of improving animals’ performance and health. To verify their impact on both in practice, it is important to test them under on-farm conditions. Therefore, the present study investigated (1) the effects of charcoal as a permanent 0.2% feed supplement and (2) the effects of an additional temporary reduction in the crude protein in the diet of turkey toms during the on-farm fattening period. The experimental conditions were standardised as far as possible; nonetheless, they were subjected to on-farm influences. Therefore, effects of the farm found in this study are not surprising, as variations are most likely for on-farm conditions influenced not only by the season but also by farm individual factors such as the supervising personnel or the respective herd.

The first aim of this study was to analyse the effects of charcoal as a 0.2% feed supplementation. Results of the present study could not confirm the positive results as found by Majewska et al. [27] and Majewska et al. [28] for body weight and mortality of turkey toms or by Kutlu, Ünsal and Görgülü [15] for the growth performance of broiler chickens from days 8 to 28 of life. These varying findings might result from the lower dose of charcoal (0.2% compared to 0.3% [24,25] or up to 10% [15]). Furthermore, the quality of charcoal is highly variable due to the production process and the original material, which affects carbon efficiency [42]. However, our results go in line with studies by Rattanawut [19] and Kana et al. [42] who also found no effects on the growth performance. Equally relevant might be the fact that these prior studies on turkeys were conducted in small groups of 34 birds each, thus providing different conditions for housing, space and infection risks than under on-farm conditions. More consistent and beneficial results have been found in ruminants [23,24] with therapeutic doses and pigs [11,12] with 0.3% dietary charcoal; both differ from poultry regarding microbial digestion. If dietary charcoal plays a role in modulating microbial flora, as it could be a secondary effect of the adsorbing characteristics, it would be interesting for further research to investigate the mechanisms of action in more detail.

Before our study, there were no results for the influence of dietary charcoal on footpad dermatitis, breast blisters or soiled cloaca in turkeys. The hypothesis was that due to the high absorption capacity of charcoal, ingested carbon could lead to drier faeces and consequently to a drier litter surface, which is known to be the highest risk factor for footpad dermatitis in poultry [1,2,43,44]. The present study found no differences in footpad lesions although the dry matter content of the litter was 6.81 percentage points higher in the treatment barns. In contrast to that, Hinz [45] showed the status of footpad dermatitis to be improved by dietary charcoal in broiler chickens. However, turkey and broiler chicken husbandry differ in housing conditions such as bedding material and fattening period. In turkey fattening, the common litter material is long-stalked straw, while in the aforementioned study by Hinz [45], the birds were kept on straw and wood pellets. Referring to previous research, straw is a more adverse bedding material compared to alternatives such as wood shavings, dried maize silage, straw pellets and rice shells. In fact, straw is inferior to other materials, especially causing litter moisture [46] and footpad dermatitis [47,48] due to its poorer water evaporation. Moreover, the fattening period of turkey toms is far longer than that of broiler chickens, predisposing turkeys to more severe lesions. On the other hand, drier litter enables the healing of the footpad lesions [49]. However, it was revealed to have no effect on either of the groups in this study and there was no evidence for a divergent development in footpad dermatitis. Breast buttons and soiled cloacae were not influenced by the diet either. Therefore, it can be concluded that feeding enriched charcoal as 0.2% feed additive does not improve the turkey’s performance or welfare status per se.

The second aim of this study was to analyse the effects of a reduction in crude protein in the animal’s diet in first and second grower feed phases. The body weight of the turkeys fed the experimental diet was reduced significantly. This was expected as protein undernutrition retards the growth rate [50,51] and breast muscle weight [31], which must be maximised in standard fattening systems by using fast growing genetics, high quality diets and efficient housing systems. An effect of interaction of diet with age was not detected. However, a numeric difference in weight between both groups could be detected in the 14th week of life, with a lower live weight of the flocks with reduced crude protein and charcoal. In the 18th week of life, with animals receiving the standard protein diet for 4 weeks, no weight difference between the control and experimental group could be detected anymore. This might be explained with the compensatory growth such as that found by Auckland and Morris [34] in turkeys, and Zubair and Leeson [35] in broiler chickens. Compensatory growth is described as increased growth after a period of undernutrition [52], and it was depicted for many different animals such as cattle [53], poultry [51,54] and fish [55]. Furthermore, the treatment group revealed a reduced mortality rate of 0.5%. This might be due to the slower growth rate, which has been reported to have a positive influence on the sudden death syndrome [56] and the prevalence of lameness [57] in broiler chickens. However, that was not investigated in the present study.

No effect of the reduced crude protein and charcoal treatment was found for the status of footpad dermatitis or for the breast blisters. Here, a higher reduction in the crude protein content in the diet could have provoked a more pronounced result. Moreover, the difference between the dry matter content of the litter in the treatment barns was smaller than in the first experiment. This was probably due to the modified diet, which could have increased the water excretion level. An increased water excretion level can be induced by a higher potassium or oligosaccharide content [2]. This coincides with the faecal quality, which was found to be better in the control group in the first half of the fattening period. Despite the lack of effects on the birds’ performance and scored health parameters, the investigated litter parameter was positively influenced. In particular, the reduced content of total nitrogen in the dry matter content could reduce the nitrogen emission of animal farming and might be worth studying further.

## 5. Conclusions

In summary, the results of the present study found no negative effects of a slight crude protein reduction in the diet on the slaughtering weight of the turkey toms. Therefore, protein reduction might be a promising approach for future studies regarding nitrogen emissions. Even if the present study found no evidence of an improved footpad health, the death rate was decreased. In the first experiment, no effects of the enriched charcoal were found. Therefore, we argue that charcoal has no influence under on-farm conditions. Yet, the presented results might be due to the combination of a protein-reduced diet supplemented with charcoal rather than to the protein reduction per se. Thus, further studies providing a protein-reduced diet without any charcoal supplementation should investigate if charcoal has an impact on the parameters studied here.

## Figures and Tables

**Table 1:** Nutrient content of the standard and reduced crude protein (rP) diets provided by the feed company for the grower and finisher feeds used in Experiments 1 and 2 for fattening the turkey toms.

Component	Unit	Experiment 1				Experiment 2					
		Grower 1	Grower 2	Grower 3	Finisher	Grower 1	Grower 1 rP	Grower 2	Grower 2 rP	Grower 3	Finisher
Metabolisable Energy	MJ/kg	12.2	12.5	12.8	13.4	12.20	12.20	12.50	12.50	12.80	13.40
Crude Protein	g/kg	230	200	170	160	230	220	200	190	170	160
Crude Fat	g/kg	61.0	57.0	65.0	94.0	62.0	62.0	64.0	59.0	70.0	91.0
Crude fibre	g/kg	32.0	30.0	30.0	30.0	31.0	30.0	29.0	28.0	31.0	31.0
Crude ash	g/kg	67.0	57.0	51.0	49.0	65.0	62.0	56.0	52.0	51.0	49.0
Methionine	g/kg	5.8	5.1	4.4	3.6	5.8	5.4	5.2	4.8	4.4	3.6
Lysine	g/kg	14.5	12.5	11.5	10.5	14.5	13.6	12.2	11.2	11.5	10.5
Ca	g/kg	10.5	9.5	8.5	8.0	10.5	9.7	9.0	8.0	8.5	8.0
P	g/kg	7.0	6.0	5.5	5.5	7.0	7.0	6.0	5.7	5.5	5.5
Na	g/kg	1.4	1.4	1.4	1.4	1.4	1.3	1.4	1.3	1.4	1.4
Lasalocid-A-sodium	mg/kg	105	-	-	-	105	105	-	-	-	-

**Table 2:** Formulae (%) provided by the feed company of the standard and protein-reduced (rP) grower and finisher feeds used in Experiments 1 and 2 for fattening the turkey toms.

Ingredient	Experiment 1				Experiment 2					
	Grower 1	Grower 2	Grower 3	Finisher	Grower 1	Grower 1 rP	Grower 2	Grower 2 rP	Grower 3	Finisher
Wheat	41.50	54.50	63.30	61.80	23.00	27.50	32.20	40.30	61.70	61.40
Soy bean extraction grist	23.60	14.20	6.70	5.60	27.60	29.50	20.50	18.00	7.60	6.10
Maize	15.00	13.00	10.00	10.00	25.00	23.00	25.00	22.00	10.00	10.00
Rape seed cake	4.00	5.00	6.00	7.00	-	-	-	-	6.00	7.00
Vegetable oils (Rapeseed, sunflower seed, palm, soy, coconut)	2.40	2.20	2.50	5.40	1.60	2.70	3.00	2.60	2.70	5.60
Sunflower seed extraction grist (peeled)	-	-	-	-	7.00	4.60	6.00	5.30	6.00	5.00
Sunflower extraction grist (partly peeled, high-protein)	8.00	5.00	6.00	5.00	-	-	-	-	-	-
Haemoglobin dried	-	1.00	-	-	1.00	-	-	-	-	-
Pork fat	1.00	1.00	1.50	1.50	1.00	-	-	-	1.80	1.00
Barley	-	-	-	-	8.00	7.40	8.00	7.00	-	-
Soybean oil	-	-	-	-	1.00	0.92	1.00	0.88	-	-

**Table 3:** Characteristics of the charcoal added to the experimental diets of the turkey toms in Experiments 1 and 2, analysed by the Fraunhofer Institute for Environmental, Safety and Energy Technology UMSICHT (Sulzbach-Rosenberg, Germany).

Cation-Exchange Capacity	Surface Area (Brunauer–Emmett–Teller Analysis)	Pore Volume	Conductivity	pH Level
17 cmol(+)/kg	263 m^2^/g	0.1692 cm^3^/g	1120 µS/cm	10.4

**Table 4:** Scoring systems used in both experiments for evaluating the health status of the turkey toms during the fattening period, and a short description of the scores.

Parameter	Source	Score	Short Description
Footpad dermatitis	Hocking et al. [38]	0	no external signs of footpad dermatitis on metatarsal pad, soft skin, no swelling or necrosis
		1	metatarsal pad harder and denser, central part of the pad
			raised, separated reticulate scales, small black necrotic areas
		2	marked swelling of the footpad, black reticulate scales, area of necrosis on less than one quarter of the footpad
		3	swelling, enlarged footpad size, pronounced, separated and more reticulate scales, necrosis on one half of the footpad
		4	as Score 3, necrosis on more than half the footpad
Breast buttons	Schulze Bisping [39]	0	intact skin, *bursa presternalis* not enlarged
		1	breast buttons <1 cm (diameter)
		2	breast buttons 1–3 cm (diameter)
		3	breast buttons >3 cm with fluctuating content
Soiled cloaca	Westermaier [40]	0	skin clean
		1	polluted with faeces
Faeces consistency (visually)	Betscher [41]	1	shaped and firm
		2	shaped and mushy
		3	unshaped and mushy
		4	watery

**Table 5:** Performance results of Experiment 1; means and standard deviation (SD) of the different parameters of the BUT6 turkey toms; of body weight (*n* = 3 farms, *n* = 6 batches, *n* = 958 turkeys (charcoal diet) diet, *n* = 960 turkeys (control)), feed intake, feed conversion ratio and mortality (*n* = 2 farms, *n* = 4 batches), litter parameter (*n* = 3 farms, *n* = 6 batches, *n* = 6 pooled samples).

Parameter	Age in Weeks	Unit	Control (Means)	SD	Charcoal Diet (Means)	SD
Body weight	6	kg	1.81	0.38	1.79	0.41
	10		5.65	0.93	5.48	0.84
	14		10.84	1.35	10.76	1.27
	18		16.39	1.70	16.59	1.79
Feed intake per bird	21 ^1^	kg	52.36	4.6	52.65	4.95
Feed conversion ratio	21 ^1^	kg/kg	2.67	0.1	2.64	0.15
Mortality	20	%	5.84	1.9	5.84	1.67
Dry matter content (litter)	21 ^1^	%	53.31	7.44	60.12	8.02
Total nitrogen (litter)	21 ^1^	% of dry matter content	4.55	0.99	3.88	0.71

^1^ after slaughtering at day 148 ± 3.

**Table 6:** Results of the scored health parameters of the BUT6 turkey toms in Experiment 1, percentage of each score per parameter (%) at the observations in weeks 6, 10, 14 and 18 of life (*n* = 3 farms, *n* = 6 batches, *n* = 918 turkeys in charcoal diet, *n* = 920 in control).

Parameter	Score	Control (Means, Incidence in %)				Charcoal Diet (Means, Incidence in %)			
Week of Life		6	10	14	18	6	10	14	18
Footpad dermatitis	0	2.75	2.08	2.08	8.96	5.56	1.88	0.21	6.04
	1	21.5	14.79	12.29	10.63	30.56	24.79	9.17	10.21
	2	62.25	74.17	70.63	45.83	55.05	70.21	68.54	50.21
	3	12.25	8.75	13.96	26.04	8.08	3.13	18.33	24.58
	4	1.25	0.21	1.04	8.54	0.76	0.00	3.75	8.96
Breast buttons	0	100.00	97.50	89.17	74.17	100.00	98.75	89.58	72.08
	1	0.00	2.50	6.25	9.58	0.00	1.25	6.67	16.25
	2	0.00	0.00	4.17	13.33	0.00	0.00	3.33	9.58
	3	0.00	0.00	0.42	2.92	0.00	0.00	0.42	2.08
Soiled cloaca	0	93.00	96.04	95.83	98.12	94.44	95.42	98.33	96.87
	1	7.00	3.96	4.17	1.88	5.56	4.58	1.67	3.13

**Table 7:** Performance results of control and charcoal + protein-reduced (rP) diets of Experiment 2, means and standard deviations (SD) of the different parameter of the BUT6 turkey toms. Body weight (*n* = 3 farms, *n* = 6 batches, *n* = 960 turkeys per group), feed intake, feed conversion ratio and mortality (*n* = 2 farms, *n* = 4 batches) and litter parameter (*n* = 3 farms, *n* = 6 batches, *n* = 6 pooled samples).

Parameter	Age in Weeks	Unit	Control (Means)	SD	Charcoal and rP Diet (Means)	SD
Body weight	6	kg	2.15	0.62	2.09	0.56
	10		5.92	1.04	5.89	1.01
	14		11.31	1.59	11.07	1.55
	18		17.67	2.11	17.54	1.94
Feed intake per bird	21 ^1^	kg	51.16	3.92	50.25	3.12
Feed conversion ratio	21 ^1^	kg/kg	2.74	0.03	2.70	0.08
Mortality	20	%	5.33	1.70	4.78	1.40
Dry matter content (litter)	21 ^1^	%	56.77	11.57	58.93	9.20
Total nitrogen (litter)	21 ^1^	% of dry matter content	3.99	0.90	3.77	0.54

^1^ after slaughtering at day 146 ± 3.

**Table 8:** Results of the scoring of health parameters of the BUT6 turkey toms in control and charcoal + protein-reduced (rP) diet groups of Experiment 2, percentage of each score per parameter (%); (*n* = 3 farms, *n* = 6 batches, *n* = 960 turkeys per group).

Parameter	Score	Control (Means, Incidence in %)				Charcoal and rP Diet (Means, Incidence in %)			
		6	10	14	18	6	10	14	18
Footpad dermatitis	0	15.63	0.42	0.83	2.71	18.75	1.04	0.83	1.04
	1	57.29	23.96	12.92	9.38	50.63	26.67	12.71	11.04
	2	25.00	59.38	59.38	56.67	25.63	60.21	61.67	56.25
	3	1.88	13.33	19.79	20.00	4.17	10.00	18.54	25.42
	4	0.21	2.92	7.08	11.25	0.83	2.08	6.25	6.25
Breast buttons	0	100.00	100.00	97.08	88.75	100.00	100.00	99.58	88.33
	1	0.00	0.00	2.08	5.83	0.00	0.00	0.42	8.75
	2	0.00	0.00	0.83	5.42	0.00	0.00	0.00	2.50
	3	0.00	0.00	0.00	0.00	0.00	0.00	0.00	0.42
Soiled cloaca	0	82.08	87.92	98.33	97.50	85.42	89.79	97.50	96.67
	1	17.92	12.08	1.67	2.50	14.58	10.21	2.50	3.33

## Appendix A

**Table A1:** Details about the periods of the batches, about the groups and the number of analysed data and about the stables and materials for manipulating in the different farms during the Experiments 1 and 2 (reduced Protein = rP).

Farm	Stables	Floor Space	Materials for Manipulating	Experiment	Batch	Groups	No. of Analysed Data (n_weight_/n_scored_)	Date
1	2	100 × 18 m	5 straw bales, 5–6 stones for pecking	1	1	Control,	160/160	03/17–07/17
						Charcoal	160/160	
					2	Control,	160/160	08/17–11/17
						Charcoal	160/160	
				2	1	Control,	160/160	11/17–03/18
						Charcoal + rP	160/160	
					2	Control,	160/160	03/18–07/18
						Charcoal + rP	160/160	
2	2	112.5 × 18 m	5 straw bales, 8 stones for pecking	1	1	Control,	160/120 ^1^	03/17–07/17
						Charcoal	160/120 ^1^	
					2	Control,	160/160	07/17–11/17
						Charcoal	160/160	
				2	1	Control,	160/160	12/17–03/18
						Charcoal + rP	160/160	
					2	Control,	160/160	03/18–07/18
						Charcoal + rP	160/160	
3	2	65 × 18 m	3 straw pellets, 6–8 white-red plastic chains	1	1	Control,	160/160	01/17–05/17
						Charcoal	158/158 ^2^	
					2	Control,	160/160	05/17–09/17
						Charcoal	160/160	
				2	1	Control,	160/160	02/18–05/18
						Charcoal + rP	160/160	
					2	Control,	160/160	06/18–10/18
						Charcoal + rP	160/160	

^1^ loss of scoring data at week 6 of age; ^2^ loss of data at week 6 of age.
